# Machine learning models for predicting preeclampsia: a systematic review

**DOI:** 10.1186/s12884-023-06220-1

**Published:** 2024-01-02

**Authors:** Amene Ranjbar, Farideh Montazeri, Sepideh Rezaei Ghamsari, Vahid Mehrnoush, Nasibeh Roozbeh, Fatemeh Darsareh

**Affiliations:** 1 Fertility and Infertility Research Center, Hormozgan University of Medical Sciences, Bandar Abbas, Iran; 2 Mother and Child Welfare Research Center, Hormozgan University of Medical Sciences, Bandar Abbas, Iran; 3 Department of Midwifery and Reproductive Health, Tehran University of Medical Sciences, Tehran, Iran

**Keywords:** Artificial intelligence, Machine learning, Preeclampsia, Systematic review

## Abstract

**Background:**

This systematic review provides an overview of machine learning (ML) approaches for predicting preeclampsia.

**Method:**

This review adhered to the Preferred Reporting Items for Systematic Reviews and Meta-Analyzes (PRISMA) guidelines. We searched the Cochrane Central Register, PubMed, EMBASE, ProQuest, Scopus, and Google Scholar up to February 2023. Search terms were limited to “preeclampsia” AND “artificial intelligence” OR “machine learning” OR “deep learning.” All studies that used ML-based analysis for predicting preeclampsia in pregnant women were considered. Non-English articles and those that are unrelated to the topic were excluded. The PROBAST was used to assess the risk of bias and applicability of each included study.

**Results:**

The search strategy yielded 128 citations; after duplicates were removed and title and abstract screening was completed, 18 full-text articles were evaluated for eligibility. Four studies were included in this review. Two studies were at low risk of bias, and two had low to moderate risk. All of the study designs included were retrospective cohort studies. Nine distinct models were chosen as ML models from the four studies. Maternal characteristics, medical history, medication intake, obstetrical history, and laboratory and ultrasound findings obtained during prenatal care visits were candidate predictors to train the ML model. Elastic net, stochastic gradient boosting, extreme gradient boosting, and Random forest were among the best models to predict preeclampsia. All four studies used metrics such as the area under the curve, true positive rate, negative positive rate, accuracy, precision, recall, and F1 score. The AUC of ML models varied from 0.860 to 0.973 in four studies.

**Conclusion:**

The results of studies yielded high prediction performance of ML models for preeclampsia risk from routine early pregnancy information.

## Background

Preeclampsia is a hypertensive disorder that usually manifests itself after 20 weeks of pregnancy, along with proteinuria [1]. It can potentially cause severe morbidity, chronic disability, and even the death of mothers and babies. Preeclampsia significantly burdens pregnant women, with an estimated incidence of 2–8% [2]. In developing countries, the prevalence of preeclampsia ranges from 1.8 to 16.7% [3]. About 12% of mothers die only from preeclampsia [4]. Because of the poorly understood causes, various risk factors, and likely multiple pathogenic phenotypes of preeclampsia, early prediction of preeclampsia is difficult. Statistical learning methods are well-equipped to deal with many variables, such as clinical and laboratory patient data, and automatically select the most informative features [5]. Artificial intelligence has been increasingly used in health and medicine in recent years. The use of artificial intelligence in obstetrics and gynecology has piqued the scientific community’s interest [6, 7].

Recent advances in computer science have propelled forward artificial intelligence. Conventional general programming algorithms generate outputs based on the input data and the rules provided, whereas artificial intelligence can create regulations and patterns based on the input and output data [8]. Artificial intelligence’s pattern recognition and prediction performance has been demonstrated in a variety of medical fields [9]. A systematic review of existing predictive models was deemed necessary to advance efforts to identify women at risk of preeclampsia as early and accurately as possible. This would allow existing models to be evaluated for their suitability for immediate use or to identify those that perform well internally but require external validation on an independent cohort before being considered for clinical use. This approach could be more efficient than adding a new model to aid in preeclampsia prevention. This systematic review aims to identify preeclampsia predictors using machine learning (ML) approaches that have been reported in previous studies in this field.

## Methods

### Study design

The Preferred Reporting Items for Systematic Reviews and Meta-Analyses (PRISMA) 2020 guidelines [10] were used to report this study.

### Objectives

To identify and summarize the predictive factors of preeclampsia using ML models and to evaluate the diagnostic accuracy of ML models in predicting preeclampsia.

**Review questions**.

1. Which ML models were used to predict preeclampsia?
2. What predictive factors of preeclampsia are used to train the ML model?
3. Which ML models had a better performance in predicting preeclampsia?
4. What is the accuracy of ML models for preeclampsia?

### Eligibility criteria

All studies that used ML-based analysis to predict preeclampsia were considered. Non-English articles and those unrelated to the topic were not considered. Letters to the editor and reviews were also excluded.

### Search strategy and selection criteria

We searched the Cochrane Central Register, PubMed, EMBASE, ProQuest, Scopus, and Google Scholar up to February 2023. Search terms were limited to “preeclampsia” AND “artificial intelligence” OR “machine learning” OR “deep learning.” Words and phrases were selected from a controlled vocabulary (MeSH, ENTREE, and others) and a free-text search for each database. In addition, the reference lists of the identified articles were also searched along with hand-searching to ensure that all documents were retrieved, which are combined using Boolean “OR” and “AND” operators. An experienced researcher searched all the databases. After eliminating duplicates, two researchers independently screened the titles and abstracts and then the full texts of potentially eligible studies against the pre-defined eligibility criteria. Consensus or an appeal to a senior researcher was used to resolve disagreements. A PRISMA flow diagram was used to document the study selection process.

### Data collection and risk of bias assessment

Data were extracted independently by two investigators. The third-party solved disagreements. The following items were extracted: (1) demographic information (the country where the data were collected, the data source, and prediction time); (2) the type of predictive model ML; and (3) the prediction results (for example, accuracy, sensitivity, true positive rate, false positive rate, specificity, and area under the recurrence curve); (4) the features used to train the ML models.

Two researchers independently assessed the quality of all included studies and discussed discrepancies until a consensus was achieved. The PROBAST [11], consisting of 20 signaling questions divided into four domains (participants, predictors, outcome, and analysis), was used to assess the risk of bias and applicability of each included study. PROBAST assists in evaluating the outcome studied by considering how it was determined, how objective it is, whether it incorporates any predictor data, how consistently it was determined across individuals, the timing of determination, whether it was independent of predictor information knowledge, and whether it matches the review question.

## Results

**Fig. 1:**
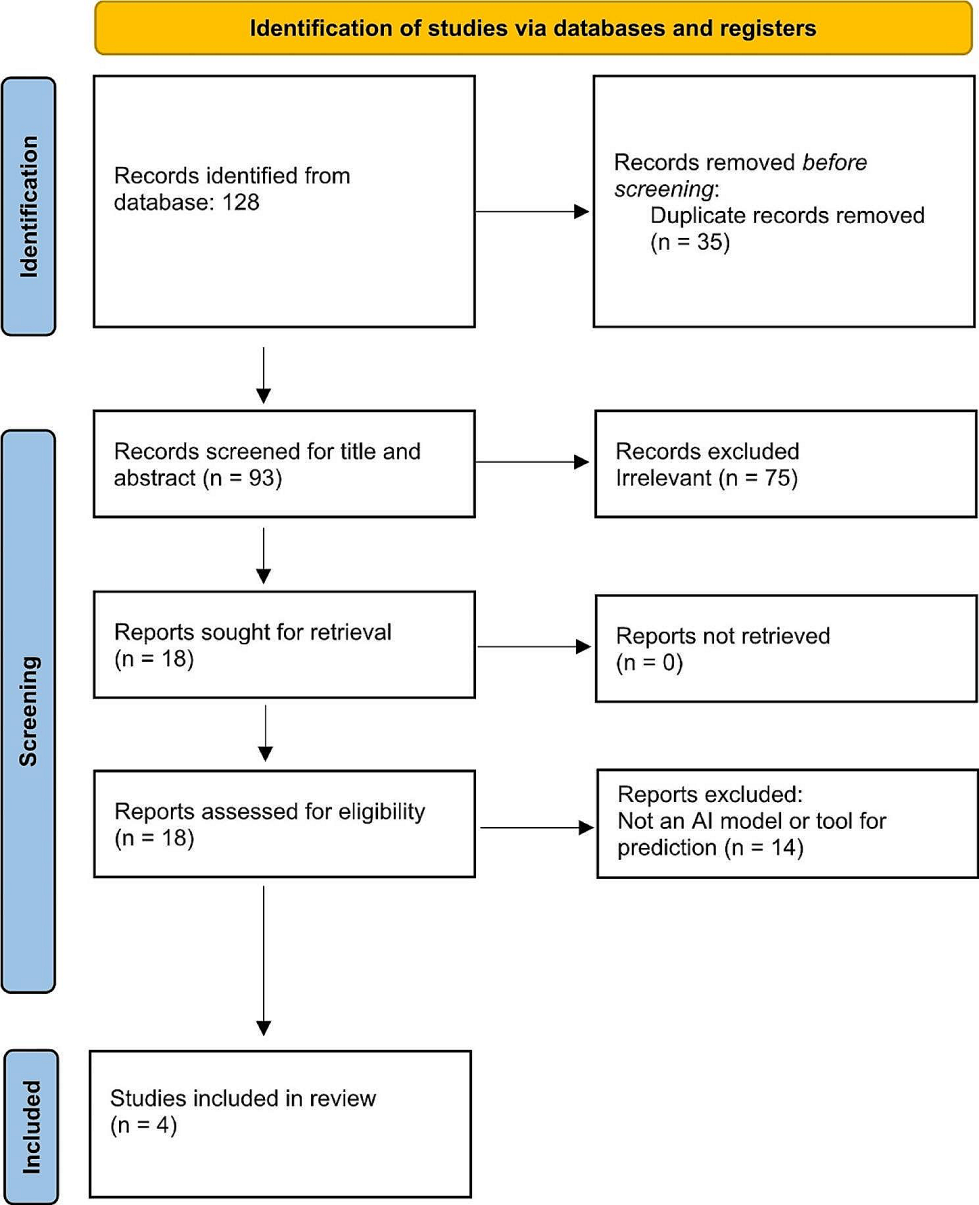
Flowchart of the study

The search strategy yielded 128 citations; after duplicates were removed and title and abstract screening was completed, 18 full-text articles were evaluated for eligibility (PRISMA Flow Diagram, Fig. 1). Four studies were included in this review. Based on the risk of bias assessment, two studies were at low risk of bias [5, 12], and two with low to moderate risk of bias [13, 14] (Table 1).

**Table 1:** PROBAST risk of bias/applicability assessment

Study	Risk of bias				Applicability			Overall	
	Participants	Predictors	Outcome	Analysis	Participants	Predictors	Outcome	Risk of bias	Applicability
1	+	+	+	+	+	+	+	+	+
2	+	+/-	+	+/-	+	+/-	+	+/-	+/-
3	+	+	+	+	+	+	+	+	+
4	+	+/-	+	+/-	+	+	+	+/-	+/-

(+) indicate low risk of bias, (+/−) indicate low/moderate risk of bias, (−) indicate high risk of bias and (?) indicate unclear risk of bias

Table 2 shows the characteristics of the included studies. The setting of the included studies was a hospital in South Korea, China, and the United States. All of the study designs included were retrospective cohort studies. Nine distinct models were chosen as ML models from the four studies (range 2–6 per study). Maternal characteristics, medical history, medication intake, obstetrical history, and laboratory and ultrasound findings obtained during prenatal care visits were candidate predictors to train the ML model. Elastic net, stochastic gradient boosting, extreme gradient boosting, and Random forest were among the best models to predict preeclampsia. All four studies used metrics such as the area under the curve, true positive rate, negative positive rate, accuracy, precision, recall, and F1 score. The AUC of ML models varied from 0.860 to 0.973 in four studies.

**Table 2:** Summary of studies

Study number	Author	Country	Data source	Prediction time	Type of ML	Findings		
						Best ML performance	Accuracy	The features used to train the model
1	Marić et al. [5]	United States	Dataset of 16,370 births	Early-onset preeclampsia (< 34 weeks gestation)	EN GB	EN	AUC: 0.890 TPR: 0.723 FPR: 0.088	Maternal characteristics Medical history Routine prenatal laboratory results Medication intake
2	Jhee et al. [13]	Republic of Korea	Dataset of 11,006 pregnant women	Late-onset preeclampsia (> 34 weeks gestation)	SGB LR DT NB SVM RF	SGB	AUC: 0.973 FPR:0.009	Maternal characteristics Medical history Laboratory results Medication intake Obstetrical history
3	Li et al. [12]	China	Dataset of 3759 pregnant women	Early-onset preeclampsia	SVM LR RF EGBM	EGBM	AUC: 0.955 Accuracy:0.920 Precision:0.447 Recall:0.789 F1 score:0.571	Maternal characteristics Medical history Laboratory results Obstetrical history
4	Liu et al. [14]	China	Dataset of 11, 472 singleton pregnancies	Not mentioned	RF SVM LR DNN DT	RF	AUC: 0.860 Accuracy:0.740 Precision:0.820 Recall:0.420 F1 score:0.560	Maternal characteristics Medical history Laboratory results Ultrasound findings Medication intake

AUC: Area under the curve; TPR: True positive rate; FPR: False positive rate; ML: Machine learning; GB: Gradient boost; EGBM: Extreme gradient boosting models; LR: Logistic regression; DT: Decision tree; DNN: Deep neural network; SVM: support vector machine; NB: naïve Bayes; RF: Random forest; EN: Elastic net; SGB: stochastic gradient boosting

## Discussion

The development of simple preeclampsia prediction methods has been a difficult task. One of the main reasons for this difficulty is that the pathogenesis of preeclampsia is complex and involves various factors [15]. Nonetheless, numerous attempts have been made to predict preeclampsia accurately, which would allow for early detection and treatment. The most common approach to increasing disease predictability has been to identify risk factors.

Parameters that have been traditionally reported to be related to preeclampsia development are previous history of preeclampsia, known chronic kidney disease, hypertension, diabetes, autoimmune disorders such as systemic lupus erythematosus and antiphospholipid syndrome, advanced maternal age (> 40 years), and a body mass index greater than 35 kg/m2 have all been linked to an increased risk of preeclampsia development [16]. Several new factors, such as Doppler and biochemical indicators, are also identified as one of the most important factors associated with developing preeclampsia [17, 18]. Most studies currently use the multiple logistic regression algorithm to predict the risk of early-onset preeclampsia or the Bayesian principle to calculate the prior risk with a simple multiple logistic regression model, then use the likelihood ratio in conjunction with special inspections to calculate the posterior risk of preeclampsia. This algorithm must frequently use different formulas to assess the risk of preeclampsia, and the included prediction indicators are often various. The British Fetal Medicine Foundation (FMF) developed and constantly improved the competitive risk model to predict preeclampsia [19]. A systematic review of preeclampsia prediction models revealed that the model’s prediction performance varies greatly. The area under the receiver operating curve varies between 0.64 and 0.96, the sensitivity ranges from 29 to 100%, and the specificity ranges from 26 to 96%, but all prediction models lack sufficient external validation [20]. However, current prediction models have drawbacks, such as a need for more model validation and limiting their clinical application. A systematic review evaluating the quality of first-trimester risk prediction models for preeclampsia found frequent methodological deficiencies in studies reporting risk prediction models for preeclampsia [21]. As a result, novel statistical approaches are urgently needed to develop an early predictive model of preeclampsia. Recently, an ML-based model was proposed as a practical antenatal preeclampsia screening method [5, 12–14].

Mari et al. used the elastic net algorithm to create a prediction model that included a subset of maternal characteristics, medical history, routine prenatal laboratory results, and medication intake. The obtained preeclampsia prediction model had an area under the curve of 0.79 (95% CI, 0.75–0.83), a sensitivity of 45.2%, and a false-positive rate of 8.1%. The early-onset preeclampsia prediction model had an area under the curve of 0.89 (95% confidence interval, 0.84–0.95), a true-positive rate of 72.3%, and a false-positive rate of 8.8% [5].

A study by Jhee et al. used the features such as maternal characteristics (age, BMI, and gestational age), maternal medical history (hypertension, diabetes, and previous preeclampsia), medications prescribed during pregnancy, biochemical laboratory data (serum blood urea nitrogen and creatinine levels, platelet counts, serum potassium level, white blood cell count, serum calcium level, and urinary protein) to train the ML models to predict the late onset of preeclampsia (Beyond the gestational age of 34 weeks). According to their findings, the area under the curve for the decision tree model, naïve Bayes classification, support vector machine, random forest algorithm, stochastic gradient boosting method, and logistic regression models were 0.857, 0.776, 0.573, 0.894, 0.924, and 0.806, respectively. The stochastic gradient boosting model performed the best in prediction accuracy and false positive rate, with values of 0.973 and 0.009, respectively [13]. The combination of maternal factors and common antenatal laboratory data from the early second to the third trimester could effectively predict late-onset preeclampsia.

In another study, Li et al. used ML models to predict the risk of preeclampsia in women using 38 candidate clinical parameters routinely available at the first visit in antenatal care, which was collected via manual chart review based on electronic health records in the early second trimester. The prediction model was built using logistic regression, random forest, support vector machine, and extreme gradient boosting. The best prediction performance was achieved by the extreme gradient boosting model (accuracy = 0.920, precision = 0.447, recall = 0.789, f1 score = 0.571, auROC = 0.955). Fasting plasma glucose was the most predictive feature of preeclampsia development, followed by mean blood pressure and body mass index [12].

Recently, Liu et al. conducted a study to develop predictive models in preeclampsia using five ML models (deep neural network, logistic regression, support vector machine, decision tree, and random forest). The feature variables to training the models included maternal characteristics (age, BMI, gestational age), medical history (previous histories of smoking, hypertension, diabetes, and previous preeclampsia), prenatal laboratory and ultrasound results (β-HCG and pregnancy-associated plasma protein A, crown-rump length, transparent layer thickness, and uterine arteries pulsatility index), and medication prescribed during pregnancy that was obtained during prenatal screening in early pregnancy. The random forest model had the highest accuracy rate compared to other prediction algorithms. The area under the curve was 0.86 (95% CI 0.80–0.92), the precision was 0.82 (95% CI 0.79–0.84), the recall rate was 0.42 (95% CI 0.41–0.44), and the F1 score was 0.56 (95% CI 0.54–0.57) [14].

According to our review, the results of studies yielded high prediction performance of ML models for predicting preeclampsia. This review will enable healthcare investigators and clinicians to consider the development or application of prediction models throughout the pregnancy period. This review shows which algorithms had demonstrated robust predictive performance for preeclampsia prediction using a similar set of predictors. Investigators in pregnancy care may also consider whether another predictive model reanalysis is required by using existing data previously analyzed by an approach that includes logistic regression. However, it should be mentioned that some of the included studies in this review reported some limitations that might lead to bias. For example in one study [13] most of the women were not included in the antenatal evaluation program until early second trimester. Therefore, first-trimester data could not be obtained. As a result, missing data or incomplete ascertainment will continue to be a limitation when applying these models in real-time with electronic health record data.

Our systematic review provided most of the evidence for developing the predictive model for Preeclampsia. In addition, it adhered to the most rigorous methodological guidelines for systematic review to ensure a high-quality review of the evidence. However, the exclusion of non-English language papers may be considered as a limitaion.

## Conclusion

The results of studies yielded high prediction performance of ML models for preeclampsia risk from routine early pregnancy information. The prediction models identified had a low to moderate risk of bias. ML can aid in a wide range of clinical domains in the obstetrical setting, and researchers should continue to investigate its vast potential.

## Data Availability

Data will be provided by the correspondence author (famadarsareh@yahoo.com) upon request.

## Abbreviations

ML: Machine learning
